# Optimization and characterization of Royal Dawn cherry (*Prunus avium*) phenolics extraction

**DOI:** 10.1038/s41598-019-54134-w

**Published:** 2019-11-26

**Authors:** Lisard Iglesias-Carres, Anna Mas-Capdevila, Francisca Isabel Bravo, Miquel Mulero, Begoña Muguerza, Anna Arola-Arnal

**Affiliations:** Nutrigenomics Research Group, Departament de Bioquímica i Biotecnología, Universitat Rovira i Virgili, 43007 Tarragona, Spain

**Keywords:** Biochemistry, Plant sciences

## Abstract

To correlate the beneficial effects of cherry consumption with their phenolic composition, a full and precise characterization is required. However, there is not a specific method to fully extract all phenolic compounds from sweet cherries. Thus, this study aimed to optimize the extraction of sweet cherry phenolics by response surface methodology and fully characterize the phenolic profile of Royal Dawn sweet cherries by HPLC-ESI-MS/MS. Extraction conditions were evaluated and optimized to 55 °C, MeOH 72%, 12 mL/g in two extraction steps. Royal Dawn sweet cherries presented rutin as the predominant phenolic compound, unlike most sweet cherry varieties. Additionally, ethanol was evaluated as a replacement solvent, obtaining lower extraction rates, especially for anthocyanins. However, in terms of total amounts, non-anthocyanin compounds were similarly extracted. The developed methodology was fast and can be routinely used in the evaluation of the phenolic profile of sweet cherries and to produce phenolic-rich extracts for the food industry.

## Introduction

Cherries are known for their wide range of bioactive compounds, including phenolic compounds^1^. The phenolic profile of sweet cherries has been widely studied^2–5^. Sweet cherries are rich in anthocyanins, hydroxycinnamic acids, flavonols and flavan-3-ols^2–5^. In sweet cherries, anthocyanins occur mostly as cyanidin-3*-O-*rutinoside^2,6^, while hydroxycinnamic acids occur mostly as chlorogenic and neochlorogenic acids^3,6,7^. Flavonols occur mainly as rutin^2,5^, and flavan-3-ols as epicatechin and catechin^2–4^.

Importantly, sweet cherry consumption has been associated with several beneficial effects^1^. To correlate its consumption with health effects, proper characterization of the phenolic profile is required. To do so, specific methodologies to fully extract phenolic compounds are necessary. In this sense, extraction factors such as temperature, liquid-to-solid ratio (LSR), solvent, and time influence the extraction of phenolic compounds from anthocyanin-rich fruits^8,9^. In the specific case of sweet cherries, several extraction parameters vary widely between studies^6,10–12^. The wide variability of extraction methods^2,4–6,11,12^ makes it controversial to compare the phenolic profile of sweet cherries among studies.

Considering the chemical complexity and variety of phenolic compounds present in fruits and vegetables^13^, as well as the factors that potentially can affect the extraction process^14^, it becomes difficult to develop a universal extraction method for all food matrices^14^. Hence, the optimization of the extraction of phenolic compounds in different food matrices is essential. In this sense, response surface methodology (RSM) has been effective to optimize polyphenols extraction from different plant materials^8,15–18^, including phenolic compounds from sour cherry pomace^8,19^. Although the phenolic profile of sour cherries is similar to the one reported for sweet cherries, relevant differences exist^20^. In this sense, the most abundant anthocyanin and flavonol in sour cherries are cyanidin-3*-O-*glucosyl-rutinoside and kaempferol-3*-O-*rutinoside, respectively^12,20,21^. Moreover, sour cherries have reported a higher total phenolic content (TPC) than that of sweet cherries^20,22^ as well as different sugar and protein contents^22^. This evidence suggests that the optimal conditions for the extraction of sweet and sour cherry phenolic compounds might differ.

To our knowledge, the only optimized extraction method for sweet cherry phenolics has been recently developed by Blackhall *et al*.^23^. However, this method was developed only to extract anthocyanins, while other relevant phenolic compounds were not considered. Indeed, the optimal extraction conditions depend on the type of phenolic compound^18^. Thus, to date, no specific methods that aim to fully extract all phenolic compounds from sweet cherries exist. Therefore, this study aimed to apply RSM to develop an extraction method that can be used to extract all phenolics present in sweet cherry varieties, and to characterize the phenolic profile of Royal Dawn sweet cherry by HPLC-ESI-MS/MS for the first time.

## Results and Discussion

Sweet cherries are a rich source of phenolic compounds with relevant biological activities^1,2^. Specific methods that fully extract phenolic compounds for each food matrix are required to completely characterize these compounds and to link food consumption with a health benefit. Methods have been developed for the extraction of anthocyanins in Lapins sweet cherries^23^ and anthocyanin-rich fruits^18,23^, and phenolic compounds from sour cherry pomace^8,19^. However, to our knowledge, no methods that aim to fully extract the most representative phenolic families of sweet cherry varieties exist. Therefore, in this study, we investigated the factors affecting sweet cherry phenolics extraction and optimized them to develop an extraction method useful in sweet cherry varieties. Specifically, the LSR, solvent percentage and extraction temperature were optimized though RSM, while extraction time and number of extractions were evaluated by classical one-variable-at-a-time approach. Methanol (MeOH) was selected as the extraction solvent thought the optimization steps of this study due to its higher extraction rate of phenolic compounds than other organic solvents^14,16,18,24^. In fact, once optimized, the extraction method was used to completely characterize by HPLC-ESI-MS/MS the phenolic profile of Royal Dawn sweet cherries for the first time. Moreover, considering the application of extraction methodologies to produce phenolic-rich extracts with potential bioactivities, ethanol (EtOH) was evaluated as MeOH replacement extraction solvent due to MeOH toxicity and prohibited use for food industry’s purposes^14^.

### Response surface methodology

The extraction of sweet cherry phenolics was optimized using the RSM approach previously used by Yılmaz *et al*. in sour cherries^8^. However, sour cherries matrix differ considerably to sweet cherries such as their most abundant phenolic compound^12,20,21^. Extraction time (30 min) was fixed during the RSM experiment in line with other studies in the literature^18,23^. The TPC, total anthocyanin content (TAC) and anthocyanins, hydroxycinnamic acids and flavonols quantified by HPLC-DAD were included in the RSM so as to predict the extraction conditions that are optimal for the most relevant phenolic families present in sweet cherries^2,3^. The experimental results for all runs were included in the model (Table 1).

**Table 1:** Rotatable central settings of independent variables and experimental results of total polyphenols content (TPC), total anthocyanins content (TAC), Cy3R (cyanidin-3-o-rutinoside), hydroxycinnamic acids (HCA) and flavonols (FO).

Run Order^a^	T (°C)	MeOH (%)	LSR (mL/g)	TPC	TAC	Cy3R	HCA	FO
1	40	100	9	5.944	1.268	3.288	8.990	0.160
2	55	80	6	6.446	1.768	3.439	9.110	0.166
3	40	0	9	5.158	0.657	1.095	7.865	0.131
4	25	80	6	5.981	1.652	3.546	9.344	0.168
5	40	50	4	5.167	1.920	3.515	8.995	0.173
6	65	50	9	7.414	1.562	3.777	7.855	0.186
7	55	20	6	5.379	0.911	2.020	7.793	0.144
8	15	50	9	7.162	1.596	3.671	9.917	0.176
9	55	80	12	8.461	1.823	4.127	11.806	0.205
10	40	50	9	5.949	1.399	3.378	9.252	0.165
11	40	50	9	6.127	1.306	2.984	8.167	0.151
12	55	20	12	7.556	1.480	3.961	11.676	0.166
13	25	20	12	7.687	1.630	4.217	12.342	0.183
14	40	50	14	6.820	1.540	3.739	11.546	0.181
15	25	20	6	5.038	0.889	1.054	6.123	0.124
16	25	80	12	7.013	1.525	3.383	11.251	0.171
17	40	50	9	6.643	1.265	2.896	7.884	0.145

Results are expressed as mg of phenolic components per gram of dry weight (mg/g dw). Abbreviations: temperature (T), methanol (MeOH), liquid-to-solid ratio (LSR). ^a^All extractions were carried out for 30 min, with 500 rpm agitation.

#### Fitting the model

The experimental data (Table 1) were used to determine the regression coefficients of Eq. (1). All the selected compounds generated a significant model, confirming that at least one of the extraction variables could explain the variation of the response variable in comparison with its mean. The coefficients of determination (R^2^) and *p*-values for the lack of fit test can be found in Table 2.

**Table 2:** Analysis of variance and regression coefficients of predicted model for response variables in sweet cherries.

Model parameters	Regression coefficient	TPC	TAC	Cy3R	HCA	FO
Intercept	β_0_	6.271	1.969	−0.044	4.957	0.161
Linear						
T	β_1_	−1.922 × 10^−1^	−4.167 × 10^−2^	−6.635 × 10^−2^	−1.039 × 10^−2^	3.811 × 10^−3^
MeOH	β_2_	2.893 × 10^−2#^	3.204 × 10^−2^*	1.100 × 10^−1^*	1.341 × 10^−2^	1.043 × 10^−2^*
LSR	β_3_	3.135 × 10^−1^*	−1.960 × 10^−1^	1.0342 × 10^−2^	4.661 × 10^−1^*	1.341 × 10^−2^*
Interaction						
T × MeOH	β_12_	4.731 × 10^−4^	1.506 × 10^−4^	−2.000 × 10^−5^	—	—
T × LSR	β_13_	1.419 × 10^−3^	2.778 × 10^−5^	−1.031 × 10^−3^	—	—
MeOH × LSR	β_23_	−2.471 × 10^−3^	−1.919 × 10^−3^*	−6.360 × 10^−3^*	—	—
Quadratic						
T × T	β_11_	2.104 × 10^−3^*	4.377 × 10^−4^	1.052 × 10^−3^	—	—
MeOH × MeOH	β_22_	−1.689 × 10^−4^	−1.372 × 10^−4#^	−3.500 × 10^−4^	—	—
LSR × LSR	β_33_	8.127×10^−4^	1.698 × 10^−2^*	2.241 × 10^−2^	—	—
R^2^		0.877	0.853	0.841	0.578	0.451
Adjusted R^2^		0.719	0.663	0.635	0.480	0.324
p-value		0.017	0.030	0.038	0.009	0.045
F-value		5.558	4.504	4.100	5.930	3.558
Lack of fit^a^		0.299	0.082	0.140	0.242	0.275

Abbreviations: temperature (T), methanol (MeOH), liquid-to-solid ratio (LSR), total polyphenol content (TPC), total anthocyanin content (TAC), Cy3R (cyanidin-3-*O*-rutinoside), hydroxycinnamic acids (HCA) and flavonols (FO). ^#^*p* < 0.1. *p < 0.05. ^a^*p*-value of lack of fit test.

#### Analysis of regression coefficients

**Figure 1:**
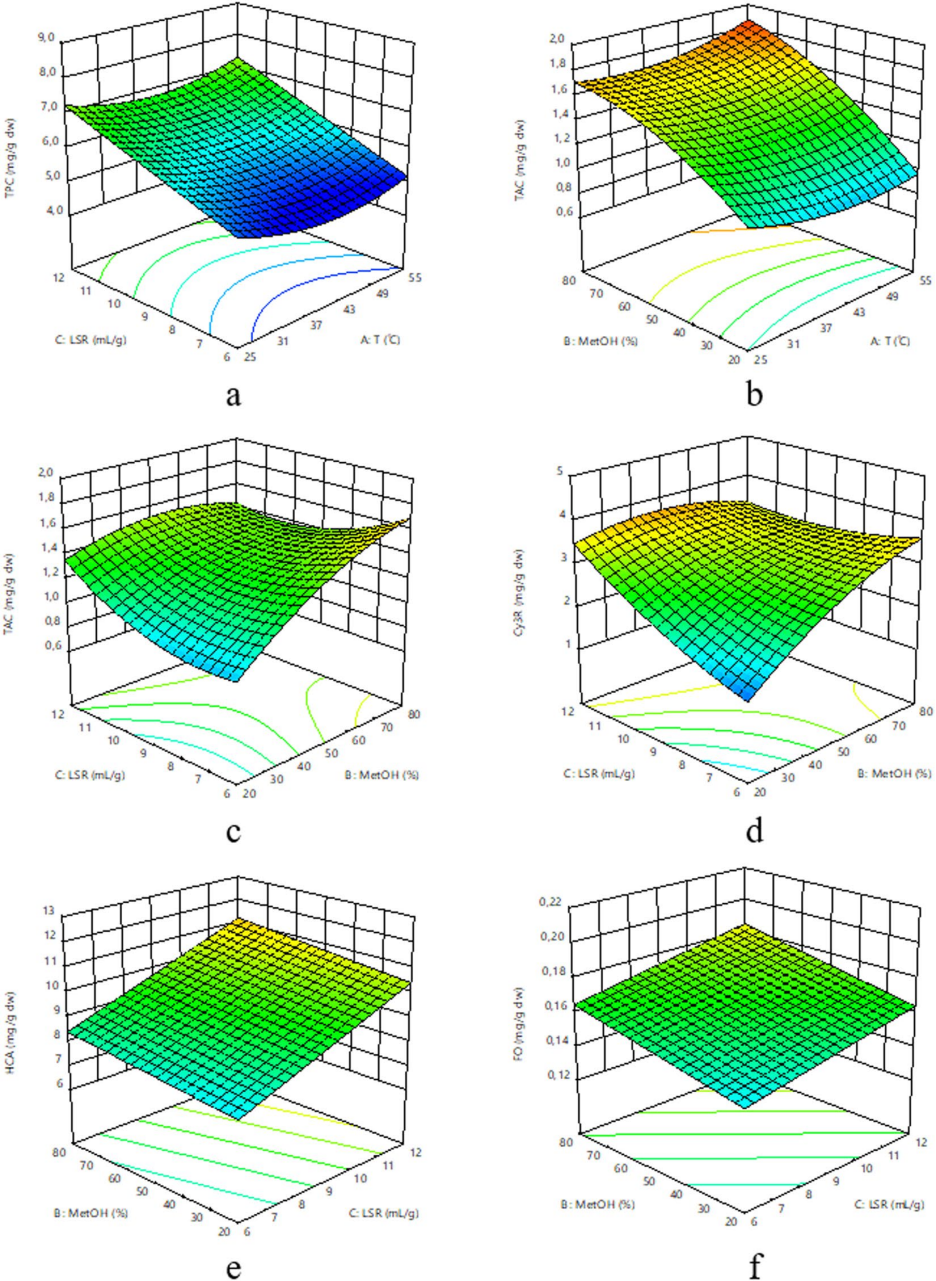
Response surface plots for total polyphenols content (TPC; **a**), total anthocyanins content (TAC; **b**,**c**), cyanidin-3-*O*-rutinoside (Cy3R; **d**), hydroxycinnamic acids (HCA; **e**) and flavonols (FO; **f**) of sweet cherries as a function of extraction temperature, methanol proportion and liquid-to-solid ratio (LSR). A at MeOH = 50%, B at LSR = 6 mL/g; and (**c**–**f** at T = 40 °C.

A significant (*p* < 0.05) positive linear effect of MeOH was found for TAC, Cy3R and FO, while a tendency (*p* < 0.1) was observed for TPC, indicating that an increase in MeOH increases the extraction of those compounds. Linear models have also been reported in the extraction of flavan-3-ols in different plant matrices^16^. A tendency (*p* < 0.1) towards negative quadratic MeOH effects was observed for the TAC, implying that its extraction increases up to an optimal MeOH percentage after which it starts to decrease (Fig. 1). Positive linear and negative quadratic effects of the extraction solvent are found for the extraction of TAC in sour cherries^8^.

No significant linear or quadratic effects were observed for TAC, Cy3R, HCA or FO, which is in disagreement with different studies in different stone and anthocyanin-rich fruits^8,17^. In agreement with our results, Ku *et al*.^25^ did not report a significant effect of temperature on the extraction of anthocyanins from *Rubus coreanus* marc. Temperature only produced a positive quadratic effect on the extraction of TPC. Similarly, the extraction of TPC, tartaric esters and flavonols from black currants was not influenced by the extraction temperature evaluated in a very similar range to our study^9^. These result suggest that the effect of temperature on the extraction of sweet cherry phenolics is not very relevant (Fig. 1), and this could be due to the maintenance of extraction temperature below 65 °C to avoid phenolics degradation^8^.

TPC, HCA and FO presented a significant positive linear effect of LSR and TAC a significant positive quadratic effect, which implies that a higher LSR will result in a higher extraction of these compounds (Fig. 1). Our results are in agreement with the extraction of different phenolic compounds from sour cherries^8^ and other plant matrices^9,16,25^. A significant interaction effect between MeOH and LSR was observed for the extraction of TAC and Cy3R, which was negative in both cases, implying that, depending on the MeOH proportion, the LSR has a different effect. Although crossover effects are not common in the literature, several studies report them^16,25^.

#### Validation of the model

The combination of extraction variables at the highest desirability (0.801) was selected to optimize the extraction method. Specifically, this corresponded to 55 °C; 72% MeOH and 12 mL/g; three extractions were performed under those conditions to confirm the model’s prediction (Table 3). No differences were obtained between the predicted and experimental values of TAC, Cy3R, HCA and FO, which confirmed the model’s accuracy. However, the TPC values were outside the range predicted by the model. However, obtaining a higher TPC than that predicted does not represent a serious drawback, as our goal was to extract the maximum phenolic compounds. Therefore, extraction temperature, MeOH concentration and LSR were fixed at 55 °C, 72% and 12 mL/g throughout the rest of the study. Surprisingly, the optimized LSR was the same as that reported for sour cherry phenolics extraction^8^ and was very similar to the one reported in the extraction of anthocyanins from Lapins sweet cherries^23^. Despite that, the MeOH concentration and extraction temperature were significantly different^8,23^.

**Table 3:** Overall optimal extraction parameters for phenolic compounds in sweet cherries.

Extraction variables				Parameters	Predicted	Experimental
T (°C)	MeOH (%)	LSR (mL/g)	Desirability			
55	72	12	0.801	TPC	7.825	10.969 ± 0.543
				TAC	1.647	1.688 ± 0.074
				Cy3R	3.808	2.953 ± 0.134
				HCA	10.944	11.979 ± 0.974
				FO	0.186	0.213 ± 0.014

Abbreviations: Temperature (T), methanol (MeOH), liquid-to-solid ratio (LSR), total polyphenol content (TPC), total anthocyanin content (TAC), Cy3R (cyanidin-3-*O*-rutinoside), hydroxycinnamic acids (HCA) and flavonols (FO). Results are expressed as mg of phenolic components per gram of dry weight (mg/g dw) ± SD (n = 3).

### Effect of time on phenolic extraction

Changes in the response variables due to the effect of time are shown in Table 4. Although different studies report a significant effect of time in anthocyanin-rich fruits^8,9,16–18,23^, in our study, no significant differences were reported due to the effect of extraction time. The fact that phenolic compounds are rapidly transferred into the extraction solvent makes our method more economically feasible than the methods developed for sour cherry phenolics (100 min) and sweet cherry anthocyanins (90 min)^8,23^. However, our results suggest that the solvent is saturated right after the sample extraction solvent are mixed, opening the door to the study of successive extractions.

**Table 4:** Effect of time on the extraction of sweet cherry phenolic compounds.

Time (min)^a^	TPC	TAC	Cy3R	HCA	FO
0	9.64 ± 1.24	1.42 ± 0.10	2.15 ± 0.23	11.67 ± 0.90	0.23 ± 0.03
20	8.98 ± 0.66	1.51 ± 0.12	2.28 ± 0.17	11.85 ± 0.76	0.22 ± 0.01
40	10.41 ± 0.45	1.43 ± 0.02	2.27 ± 0.04	12.23 ± 0.37	0.24 ± 0.02
60	9.99 ± 0.23	1.43 ± 0.05	2.35 ± 0.19	12.72 ± 0.50	0.24 ± 0.02
80	10.42 ± 0.16	1.42 ± 0.08	2.31 ± ± 0.13	12.25 ± 0.33	0.25 ± 0.01
100	9.41 ± 0.72	1.37 ± 0.13	2.27 ± 0.03	12.60 ± 0.31	0.25 ± 0.04
120	9.23 ± 1.54	1.37 ± 0.11	2.20 ± 0.26	12.19 ± 1.81	0.23 ± 0.05

Results are expressed as mg of phenolic components per gram of dry weight (mg/g dw) ± SD (n = 3). *p*-values for all parameters were higher than 0.05 by a one-way ANOVA (Tukey’s test). Abbreviations: total polyphenol content (TPC), total anthocyanin content (TAC), Cy3R (cyanidin-3-*O*-rutinoside), hydroxycinnamic acids (HCA) and flavonols (FO).

### Effect of multiple-step extractions on phenolic extraction

**Figure 2:**
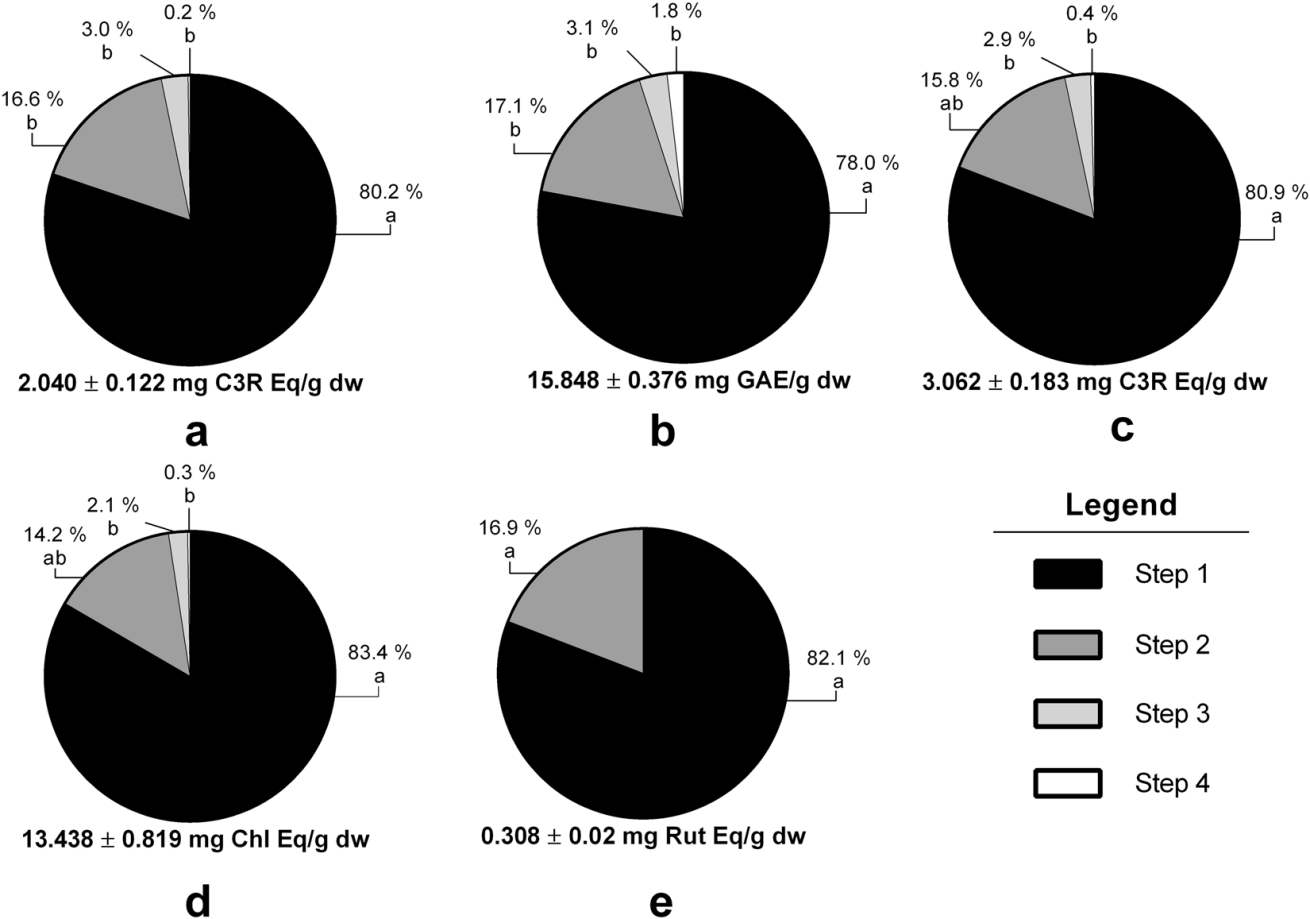
Effect of sequential extraction on the extraction of total polyphenols content (TPC; **a**), total anthocyanins content (TAC; **b**), cyanidin-3-*O*-rutinoside (Cy3R; **c**), hydroxycinnamic acids (HCA; **d**) and flavonols (FO; **e**) from sweet cherries. The results are expressed as milligrams of phenolic equivalent per gram of dry weigh ± SD (n = 3) and percentage. Different letters (one-way ANOVA, Tukey’s test) indicate significant differences between extraction steps.

Multi-step extractions are a useful strategy to increase the extraction yield of phenolic compounds in food matrices^15^. The results show a considerable increase in the extraction of phenolic compounds between the first and second extraction steps (Fig. 2). However, after the second extraction step, no significant increases were found, indicating that the extraction is mostly completed at the second extraction step. Therefore, two sequential steps were defined as optimal and used throughout the rest of the experiment.

### Phenolic profile of royal dawn sweet cherries by HPLC-ESI-MS/MS

The phenolic profile of Royal Dawn sweet cherries by HPLC-ESI-MS/MS (Table 5) is in agreement with the major phenolic families occurring in other sweet cherries^2–5^. Cyanidin-based anthocyanins were found to be predominant, and cyanidin-3*-O-*rutinoside was the anthocyanin with the highest concentration, which is consistent with the literature^2,3,10,12^. Several hydroxycinnamic acids were found in high concentrations in this study, which is consistent with the fact that stone fruits are rich in these type of phenolic compounds^7^. Indeed, caffeoylquinic acid derivatives are widely reported among the phenolic compounds with the highest concentration in sweet cherries. Rutin was found at a higher concentration than that of any other compound in our study, and although rutin is reported as the main flavonol in sweet cherries^2,5^, only few varieties report it as the predominant phenolic compound^6,10^. The main flavan-3-ol representative in Royal Dawn sweet cherries was epicatechin, which had a 3-fold higher concentration than that of catechin and this follows the general trend that epicatechin is more concentrated than catechin in sweet cherries^2,5^. Procyanidin dimer B2 was the procyanidin with the highest concentration and reached higher concentrations than those of catechin. Although not common, this trend has been observed in other varieties such as Beritello sweet cherries^5^.

**Table 5:** Phenolic compounds of sweet cherry extracted using methanol (MeOH) or ethanol (EtOH) as extraction solvents.

Compound	MeOH	EtOH	*p*-Value
Benzoic acid	2.39 ± 0.17	2.23 ± 0.04	0.20
Phloroglucinol	n.d.	n.d.	
Hydroxybenzoic acid^a^	0.75 ± 0.02	0.81 ± 0.00	0.01
Dihydroxybenzoic acid^b^	0.32 ± 0.00	0.33 ± 0.02	0.71
Protocatechuic acid	1.94 ± 0.04	2.90 ± 0.21	<0.01
*p*-Coumaric acid	0.12 ± 0.00	0.11 ± 0.00	<0.01
Gallic acid	0.02 ± 0.01	0.02 ± 0.01	0.98
Caffeic acid	0.40 ± 0.01	0.38 ± 0.00	0.07
Ferulic acid	0.36 ± 0.01	0.29 ± 0.01	<0.01
Resveratrol	0.30 ± 0.13	0.11 ± 0.05	0.08
Apigenin	0.04 ± 0.00	0.04 ± 0.01	0.92
Kempferol	0.02 ± 0.00	n.q.	
Eriodictyol	0.06 ± 0.02	0.05 ± 0.02	0.26
Catechin	16.36 ± 0.46	18.49 ± 2.77	0.26
Epicatechin	54.77 ± 0.57	46.46 ± 1.51	<0.01
Quercetin	1.55 ± 0.10	4.03 ± 0.19	<0.01
Caffeoyltartaric acid^c^	2.32 ± 0.11	2.75 ± 0.05	<0.01
Isorhamnetin	3.72 ± 0.08	3.80 ± 0.07	0.26
*p*-coumaric acid *O*-glucoside d1^d^	0.91 ± 0.03	0.99 ± 0.02	0.01
*p*-coumaric acid *O*-glucoside d2^d^	0.23 ± 0.00	0.22 ± 0.02	0.41
*p*-coumaric acid *O*-glucoside d3^d^	0.48 ± 0.01	0.46 ± 0.04	0.68
*p*-coumaric acid *O*-glucoside d4^d^	5.01 ± 0.15	5.76 ± 0.09	<0.01
*p*-coumaric acid *O*-glucoside d5^d^	0.54 ± 0.01	0.52 ± 0.03	0.31
Gallic acid *O*-glucoside d1^e^	0.03 ± 0.00	0.03 ± 0.00	0.65
Gallic acid *O*-glucoside d2^e^	0.11 ± 0.00	0.11 ± 0.00	0.20
Caffeic acid *O*-glucoside^c^	241.95 ± 4.15	276.90 ± 7.12	<0.01
Neochlorogenic acid^f^	263.42 ± 32.21	235.01 ± 43.60	0.42
Chlorogenic acid	111.84 ± 5.94	89.87 ± 28.31	0.26
Cryptogenic acid^f^	34.81 ± 0.19	32.86 ± 1.77	0.13
Feruloylquinic acid^g^	1.66 ± 0.05	1.79 ± 0.04	0.02
Resveratrol *O*-glucoside d1^h^	0.37 ± 0.02	0.32 ± 0.01	0.01
Resveratrol *O*-glucoside d2^h^	0.52 ± 0.12	0.40 ± 0.01	0.15
Kaempferol-3-*O*-glucoside	2.55 ± 0.11	2.14 ± 0.04	<0.01
Eriodictyol-7-*O*-glucoside	0.40 ± 0.05	0.38 ± 0.16	0.85
Catechin *O*-glucose^i^	0.18 ± 0.01	0.21 ± 0.02	0.07
EGCG	0.04 ± 0.00	0.05 ± 0.00	<0.01
Quercetin *O*-glucoside^j^	13.11 ± 0.17	10.39 ± 0.25	<0.01
Hyperoside	n.q.	n.q.	
Isorhamnetin-3-*O*-glucoside	0.16 ± 0.03	0.11 ± 0.01	0.03
Procyanidin dimer d1^k^	6.25 ± 0.10	7.27 ± 1.35	0.26
Procyanidin dimer B2	44.15 ± 0.43	39.34 ± 1.73	0.01
Procyanidin dimer d2^k^	2.80 ± 0.28	2.54 ± 0.36	0.38
Procyanidin dimer d3^k^	6.07 ± 0.16	4.74 ± 0.21	<0.01
Kaempferol-3-*O*-rutinoside	46.22 ± 0.50	39.45 ± 0.61	<0.01
Rutin	2141.34 ± 125.08	2194.54 ± 7.54	0.41
Procyanidin trimer^k^	1.63 ± 0.02	1.34 ± 0.07	<0.01
Cyanidin *O*-arabinoside^l^	13.93 ± 0.60	2.09 ± 0.23	<0.01
Cyanidin *O*-caffeoylglucose d1^l^	0.37 ± 0.03	0.12 ± 0.01	<0.01
Cyanidin *O*-caffeoylglucose d2^l^	7.78 ± 0.36	1.09 ± 0.04	<0.01
Cyanidin *O*-glucose d1^l^	213.83 ± 41.4	22.21 ± 2.31	<0.01
Cyanidin *O*-glucose d2^l^	3.13 ± 0.09	0.31 ± 0.03	<0.01
Cyanidin-3-*O*-rutinoside	942.91 ± 170.29	29.21 ± 3.41	<0.01
Delphinidin 3-*O*-rutinoside^l^	0.14 ± 0.01	n.q.	
Delphinidin *O*-coumaroylglucose d1^l^	0.96 ± 0.07	9.30 ± 0.26	<0.01
Delphinidin *O*-coumaroylglucose d2^l^	9.91 ± 0.16	5.03 ± 0.08	<0.01
Delphinidin *O*-coumaroylglucose d3^l^	97.61 ± 20.18	46.37 ± 0.82	0.02
Malvidin *O*-coumaroylglucose^m^	n.q.	0.04 ± 0.01	
Malvidin-3-*O*-glucoside	0.36 ± 0.12	0.50 ± 0.25	0.514
Pelargonidin *O*-glucose d1^l^	7.81 ± 0.11	0.41 ± 0.04	<0.01
Pelargonidin O-glucose d2^l^	n.q.	0.37 ± 0.04	
Peonidin-3-*O*-rutinoside	32.97 ± 1.48	5.26 ± 0.20	<0.01

Results are expressed in mg/kg dw ± SD (n = 3). Statistics by Student’s t-test. d1, d2, d3, d4 and d5 indicate different isomeric compounds. ^a^Quantified using the calibration curve of benzoic acid. ^b^Quantified using the calibration curve of protocatechuic acid. ^c^Quantified using the calibration curve of caffeic acid. ^d^Quantified using the calibration curve of *p*-coumaric acid. ^e^Quantified using the calibration curve of gallic acid. ^f^Quantified using the calibration curve of chlorogenic acid. ^g^Quantified using the calibration curve of ferulic acid. ^h^Quantified using the calibration curve of resveratrol. ^i^Quantified using the calibration curve of catechin. ^j^Quantified using the calibration curve of hyperoside. ^k^Quantified using the calibration curve of procyanidin dimer B2. ^l^Compounds quantified using the calibration curve of cyanidin-3-*O*-rutinoside. ^m^Compounds quantified using the calibration curve of malvidin-3-*O*-glucoside. Abbreviations: n.d., not detected, n.q., not quantified.

### Investigation of solvent replacement

The solvent EtOH was included in the study to evaluate the potential of the developed method to generate phenolic-rich extracts for the food industry. The extraction conditions were the same as the optimized in MeOH (two consecutive extractions, 55 °C, 72% and 12 mL/g). The methanolic and ethanolic extracts of sweet cherries showed that, in general, phenolic compounds were better extracted in MeOH than they were in EtOH (Table 5), which is consistent with the literature^14,16,18,24^. In the specific case of anthocyanins, methanolic extraction achieved significantly higher yield, which were also relevant in terms of total amounts. Only a few anthocyanins (i.e., delphinidin *O*-coumaroylglucose d1) were extracted at higher amounts in the ethanol-based extraction. Consistent with our results, MeOH was a better extraction solvent for anthocyanins in blueberries^24^. For the non-anthocyanin compounds, MeOH based-extraction only achieved statistically significant and relevant higher extraction rates (>20%) of ferulic acid, quercetin-*O*-glucoside, isorhamnetin-3*-O-*glucoside, procyanidin dimer d3 and procyanidin trimer. For the ethanol-based extraction, only protocatechuic acid and quercetin, which were significantly extracted in higher amounts with EtOH, reached a relevant increase (>20%) of their concentration. Our results are in agreement with other studies that evaluate the extraction of non-anthocyanin phenolic compounds in sour cherry pomace^8^. With the exception of anthocyanins, relevant sweet cherry phenolics with potential bioactivities^10,13^, such as rutin or procyanidin dimer B2, were similarly extracted in both extraction solvents. Consequently, the adaptation of ethanol-based extraction to the food industry could still be useful to produce phenolic extracts with potential health bioactive effects. Additionally, the use of MeOH-based methodology can be used to routinely characterize phenolic profiles from sweet cherries.

We optimized by RSM a specific method to rapidly extract all phenolic compounds from sweet cherries. Additionally, we used the optimized method to fully extract and correctly profile by HPLC-ESI-MS/MS the phenolic composition of Royal Dawn sweet cherries and demonstrated that, unlike most sweet cherry varieties, rutin is the predominant phenolic compound. This methodology could be routinely used to extract phenolics from sweet cherries for their full characterization. This characterization is essential to link cherry fruit consumption health-promoting effects with their phenolic profile. Moreover, this method could be applied to produce phenolic-rich extracts for the food industry.

## Materials and Methods

### Plant material

Royal Dawn sweet cherries (*Prunus avium*) were purchased from Mercabarna (Barcelona, Spain) and were originally from Mendoza (Argentina). Cherry stones were manually removed and flesh was frozen in liquid nitrogen and grounded. Next, homogenates were lyophilized for a week in a Telstar LyoQuest lyophilizer (Thermo Fisher Scientific, Madrid, Spain) at −55 °C and ground to a fine homogeneous powder using a conventional chopping machine (Moulinette 1, 2, 3, Moulinex) which was kept dry and protected from humidity and light exposure until extraction.

### Chemicals and reagents

All water used in this study was ultrapure water, which was obtained from a Milli-Q Advantage A10 system (Madrid, Spain). The organic solvents used for the HPLC analyses and the extraction of phenolic compounds from sweet cherries (acetonitrile, ethanol and methanol) as well as glacial acetic acid were all HPLC analytical grade and were purchased from Panreac (Barcelona, Spain). Formic acid was purchased from Scharlab (Barcelona, Spain). The Folin-Ciocalteu reagent was purchased from Fluka/Sigma-Aldrich (Madrid, Spain). The standard compounds apigenin, chlorogenic acid, eriodictyol, eriodyctiol-7*-O-*glucoside, hyperoside (quercetin-3*-O-*glucoside), isorhamnetin, isorhamnetin-3*-O-*glucoside, kaempferol, kaempferol-3*-O-*glucoside, and kaempferol-3*-O-*rutinoside were purchased from Extrasynthese (Lyon, France). The standard compounds benzoic acid, caffeic acid, (+)-catechin, epigallocatechin gallate (EGCG), *p*-coumaric acid, (−)-epicatechin, ferulic acid, gallic acid, phloroglucinol, procyanidin dimer B2, protocatechuic acid and quercetin were purchased from Fluka/Sigma-Aldrich (Barcelona, Spain). The standard anthocyanin compounds cyanidin-3-*O*-rutinoside, malvidin-3-*O*-glucoside and peonidin-3-*O*-rutinoside were purchased from PhytoLab (Vestenbergsgreuth, Germany). Resveratrol was purchased from Quimivita (Barcelona, Spain), and rutin was kindly provided by Nutrafur (Murcia, Spain).

To conduct this study, all non-anthocyanidin standard compounds were dissolved individually in MeOH at 2 mg/mL, with the exception of isorhamnetin-3*-O-*glucoside (1 mg/mL) and hyperoside (0.5 mg/mL). Anthocyanidin standard compounds (cyanidin-3*-O-*rutinoside, malvidin-3*-O-*glucoside and peonidin-3*-O-*rutinoside) were dissolved individually in MeOH (0.01% HCl) at 0.5 mg/mL. These standard stock solutions were stored in amber glass flasks at −20 °C and prepared newly when older than 3 month and used to construct calibration curves for polyphenols quantification.

### Extraction procedure

Cherry powder was weighed to obtain the desired LSR and mixed with 1.5 mL of pre-heated extraction solvent (methanol:water, v:v). Different extraction MeOH concentrations, extraction temperatures, times and extraction steps were used throughout the experiment. MeOH was prepared in all cases including 1% formic acid to promote plant’s matrix degradation^16^. Extractions were performed in 2 mL Eppendorf tubes in a shaking and heating plate (Thermo Fischer Scientific, Madrid, Spain) at 500 rpm agitation under protection from light exposure and then samples were centrifuged at 9,500 g for 10 min at 4 °C. Supernatants were stored at −20 °C until further analyses.

### Response surface design

The extraction of sweet cherry phenolics was optimized using an experimental design by RSM^8^. A rotatable central composite design with three factors and five levels was selected. The design consisted of 17 randomized runs with three center point replicates. The independent variables used were temperature (T, X_1_; 15–65 °C), methanol concentration (MeOH, X_2_; methanol:water, 0–100%) and LSR (X_3_; 4–14 mL/g). Extraction time (30 min) was fixed as a constant during the RSM experiment. Experimental data were fitted to a second polynomial response surface, which follows the equation:

1 $$y={\beta }_{0}+{\sum }_{i=1}^{k}{\beta }_{i}{X}_{i}+{\sum }_{i=1}^{k}{\beta }_{ii}{X}_{i}^{2}+{\sum }_{\begin{array}{c}i=1\\ i < j\end{array}}^{k-1}{\sum }_{j=2}^{k}{\beta }_{ij}{X}_{i}{X}_{j}$$

where Y is the dependent variable, β_0_ the constant coefficient, and β_i_, β_ii_ and β_ij_ are the linear, quadratic and interaction regression coefficients, respectively. X_i_, X_ii_ and X_ij_ represent the independent variables. Independent variables included generic determinations and individual compounds detected by HPLC-DAD. The results of the RSM design were analyzed with Design-expert 9.0.6 software (Trial version, Stat-Ease Inc., Minneapolis, MN, USA).

### Kinetic study

A kinetic study was performed to evaluate the effect of time on the polyphenols extraction yield in sweet cherries. Seven extraction times from 0 to 120 min were selected. The LSR was fixed at 12 mL/g, MeOH percentage at 72% and temperature at 55 °C. The TPC, TAC and anthocyanins, hydroxycinnamic acids and flavonols quantified by HPLC-DAD were used to evaluate the effect of time on polyphenols extractability.

### Effect of multi-step extractions

Four consecutive extractions were performed in order to evaluate the influence of multiple extractions on polyphenols extraction yield in sweet cherries. Samples were mixed with the pre-heated (55 °C) extraction solvent (MeOH of 72%) in a LSR of 12 mL/g and immediately centrifuged (9,500 × g, 10 min, 4 °C). Pellets were re-extracted under the same extraction conditions three more times, and supernatants were collected again and stored for polyphenols content analyses. The TPC, TAC and anthocyanins, hydroxycinnamic acids and flavonols quantified by HPLC-DAD were used to evaluate the effect of sequential extractions on the polyphenols extraction yield.

### Phenolic characterization of sweet cherries

Sweet cherry phenolic profile was accurately quantified in methanol- and an ethanol-based (EtOH) extractions. Briefly, samples were mixed with the pre-heated (55 °C) extraction solvent (MeOH or EtOH of 72% including 1% formic acid) in a LSR of 12 mL/g and immediately centrifuged (9,500 g, 10 min, 4 °C). This procedure was conducted twice, and supernatants were recollected and analyzed. The characterization of sweet cherries was performed by the developed HPLC-ESI-MS/MS methodology.

### Analysis of response variables

#### Total polyphenol and anthocyanin contents

The TPC and TAC of cherry extracts were determined by the Folin-Ciocalteu and pH differential methods from Iglesias-Carres *et al*.^18^. The results were expressed as milligram of gallic acid or cyanidin-3*-O-*rutinoside equivalent per gram of dry weight (mg GAE or Cy3R/g dw). The molar absorbance of Cy3R (595.2 g/mol) used was 28,800 L/mol × cm.

#### HPLC-DAD and HPLC-ESI-MS/MS quantification of phenolic compounds

In the RSM study, the detection and quantification of sweet cherry phenolics was performed by HPLC-DAD in the same system and conditions developed in Iglesias-Carres *et al*.^18^. Method quality parameters can be found in S1 Table.

In the HPLC-ESI-MS/MS quantification system, the extracts were directly analyzed using a 1200 LC Series coupled to a 6410 MS/MS (Agilent Technologies, Palo Alto, CA, USA). Of note, two different HPLC-ESI-MS/MS systems were used to separate, detect and quantify non-anthocyanin and anthocyanin phenolic compounds. Non-anthocyanin compounds separation was achieved using a ZORBAX Eclipse XDB-C18 (150 mm × 2.1 mm i.d., 5 µm particle size) as the chromatographic column equipped with a Narrow-Bore guard column (2.1 mm × 12.5 mm, 5 µm particle size) (Agilent Technologies, Palo Alto, CA, USA) as previously described in Iglesias-Carres *et al*.^18^. Separation of anthocyanins was achieved using an Acquity BHE C18 column (50 mm × 2.1 mm, 5 µm particle size) (Waters, Milford, MA, USA) as previously described in Iglesias-Carres *et al*.^18^. Optimized conditions for the analysis of non-anthocyanin and anthocyanin phenolic compounds are summarized in S2 Table. In both methodologies, data acquisition was carried out using MassHunter Software (Agilent Technologies, Palo Alto, CA, USA). The calibration curves, coefficient of determination, linearity and detection and quantification limits for non-anthocyanin and anthocyanin phenolic compounds can be found in S3 Table.

### Statistical analysis

All experiments carried out thought this manuscript were performed in triplicates. Design-expert 9.0.6 software (Trial version, Stat-Ease Inc., Minneapolis, MN, USA) was used to analyze the results of the RSM section. For any other statistical analysis SPSS 19 software (SPSS Inc., Chicago, IL, USA) was used. The statistics’ significance was evaluated using a one-way ANOVA (Tukey’s test) or Student’s t-test, and statistical significance was considered when *p* < 0.05.

### Abbreviations

Cy3R, cyanidin-3-*O*-rutinoside; dw, dry weigh; EtOH, ethanol; FO, flavonols; GAE, gallic acid equivalents; HCA, hydroxycinnamic acids; LSR, liquid-to-solid ratio; MeOH, methanol; T, temperature; TAC, total anthocyanin content; and TPC, total polyphenol content.

## Supplementary information

Supplementary information

## Data Availability

All data generated or analyzed during this study are included in this published article (and its Supplementary Information Files).

## References

[1] Ferretti G, Bacchetti T, Belleggia A, Neri D (2010). Cherry Antioxidants: From Farm to Table. Molecules.

[2] Martini S, Conte A, Tagliazucchi D (2017). Phenolic compounds profile and antioxidant properties of six sweet cherry (*Prunus avium*) cultivars. Food Res. Int..

[3] Chockchaisawasdee S, Golding JB, Vuong QV, Papoutsis K, Stathopoulos CE (2016). Sweet cherry: Composition, postharvest preservation, processing and trends for its future use. Trends Food Sci. Technol..

[4] Wang M, Jiang N, Wang Y, Jiang D, Feng X (2017). Characterization of Phenolic Compounds from Early and Late Ripening Sweet Cherries and Their Antioxidant and Antifungal Activities. J. Agric. Food Chem..

[5] Di Matteo A, Russo R, Graziani G, Ritieni A, Di Vaio C (2017). Characterization of autochthonous sweet cherry cultivars (*Prunus avium L*.) of southern Italy for fruit quality, bioactive compounds and antioxidant activity. J. Sci. Food Agric..

[6] Usenik V, Fabcic J, Stampar F (2008). Sugars, organic acids, phenolic composition and antioxidant activity of sweet cherry (*Prunus avium L*.). Food Chem..

[7] Redondo D, Arias E, Oria R, Venturini ME (2017). Thinned stone fruits are a source of polyphenols and antioxidant compounds. J. Sci. Food Agric..

[8] Yılmaz FM, Karaaslan M, Vardin H (2015). Optimization of extraction parameters on the isolation of phenolic compounds from sour cherry (*Prunus cerasus L*.) pomace. J. Food Sci. Technol..

[9] Cacace JE, Mazza G (2003). J.1365-2621.2003.Tb14146.X. J. Food Sci..

[10] Kelebek H, Selli S (2011). Evaluation of chemical constituents and antioxidant activity of sweet cherry (*Prunus avium L*.) cultivars. Int. J. Food Sci. Technol..

[11] Paul KM, Plucker JA (2004). Two steps forward, one step back: Effect size reporting in gifted education research from 1995-2000. Roeper Rev..

[12] Chemistry F (2004). Anthocyanin and Polyphenolic Composition. J. Food Sci..

[13] Del Rio D (2013). Dietary (Poly)phenolics in Human Health: Structures, Bioavailability, and Evidence of Protective Effects Against Chronic Diseases. Antioxid. Redox Signal..

[14] Kushwaha R, Karanjekar S (2011). Standardization of ashwagandharishta formulation by TLC method. Int. J. ChemTech Res..

[15] Yang L (2009). Optimum extraction process of polyphenols from the bark of *Phyllanthus emblica L*. based on the response surface methodology. J. Sep. Sci..

[16] Borges GDSC, Vieira FGK, Copetti C, Gonzaga LV, Fett R (2011). Optimization of the extraction of flavanols and anthocyanins from the fruit pulp of *Euterpe edulis* using the response surface methodology. Food Res. Int..

[17] Pompeu DR, Silva EM, Rogez H (2009). Optimisation of the solvent extraction of phenolic antioxidants from fruits of *Euterpe oleracea* using Response Surface Methodology. Bioresour. Technol..

[18] Iglesias-Carres L (2018). Optimized Extraction by Response Surface Methodology Used for the Characterization and Quantification of Phenolic Compounds in Whole Red Grapes (*Vitis vinifera*). Nutrients.

[19] Elez Garofulić I, Dragović-Uzelac V, Režek Jambrak A, Jukić M (2013). The effect of microwave assisted extraction on the isolation of anthocyanins and phenolic acids from sour cherry Marasca (*Prunus cerasus* var. Marasca). J. Food Eng..

[20] Kim DO, Ho JH, Young JK, Hyun SY, Lee CY (2005). Sweet and sour cherry phenolics and their protective effects on neuronal cells. J. Agric. Food Chem..

[21] Wojdyło A, Nowicka P, Laskowski P, Oszmiański J (2014). Evaluation of sour cherry (*Prunus cerasus L*.) fruits for their polyphenol content, antioxidant properties, and nutritional components. J. Agric. Food Chem..

[22] McCune LM, Kubota C, Stendell-Hollis NR, Thomson CA (2011). Cherries and Health: A Review. Crit. Rev. Food Sci. Nutr..

[23] Blackhall ML, Berry R, Davies NW, Walls JT (2018). Optimized extraction of anthocyanins from Reid Fruits’ *Prunus avium* ‘Lapins’ cherries. Food Chem..

[24] Silva S, Costa EM, Calhau C, Morais RM, Pintado MME (2017). Production of a food grade blueberry extract rich in anthocyanins: selection of solvents, extraction conditions and purification method. J. Food Meas. Charact..

[25] Ku CS, Mun SP (2008). Optimization of the extraction of anthocyanin from Bokbunja (*Rubus coreanus* Miq.) marc produced during traditional wine processing and characterization of the extracts. Bioresour. Technol..

