# Supplementary material for: Optimization and characterization of Royal Dawn cherry (Prunus avium) phenolics extraction
Source: Sci Rep. 2019 Nov 26;9:17626. doi: 10.1038/s41598-019-54134-w (PMC6879531; doi:10.1038/s41598-019-54134-w)
Supplement: Supplementary file 1 — Supplementary information [file 41598_2019_54134_MOESM1_ESM.pdf]

Table S1: Retention time (RT), detection wavelength, calibration curves, determination coefficient (R^2), linearity range, method precision, method reproducibility, limit of detection (LOD) and limit of quantification (LOQ) for phenolic compounds detected by the HPLC-DAD method.

Compound	RT (min)	Detection Wavelength (nm)	Calibration Curve	R^2	Linearity (µM)	Precision (% RSD, n=3)			reproducibility (% RSD, n=3)			LOD (µM)	LOQ (µM)
						100 µg/mL	50 µg/mL	1 µg/mL	100 µg/mL	50 µg/mL	1 µg/mL		
Chlorogenic acid	7.758	320	y=26.295x	0.9995	2.822 - 141.119	0.128	0.018	0.193	0.465	0.192	0.308	0.163	0.543
Cyanidin-3-*O*-rutinoside	14.35	520	y=47.880x	0.9990	1.679 - 83.959	0.092	0.176	0.269	0.506	0.260	0.471	0.006	0.021
Rutin	19.74	340	y=29.296x	0.9992	0.164 - 81.897	0.095	0.026	0.206	0.525	0.185	0.361	0.018	0.060

Table S2: Molecular weight, exact mass identified by qTOF and quantification conditions for the quantification of anthocyanins in sweet cherries by HPLC-ESI-MS/MS.

Compound[a]	MW	$[M-H]^-$ or $[M]^{+b}$	Quantification		Confirmation	
			MS/MS	CE (V)	MS/MS	CE (V)
Benzoic acid	122.12	121.0304	121>77	8	121>59	4
Phloroglucinol	126.11	125.0252	125>57	20	125>125	0
Hydroxybenzoic acid	138.12	137.0256	137>93	40	137>65	36
Protocatechuic acid	154.12	153.0217	153>109	16	153>62	40
Dihydroxybenzoic acid	154.12	153.0215	153>109	16	153>62	40
p-Coumaric acid	164.16	163.0774	163>119	16	163>93	36
Gallic Acid	170.12	169.0137	169>125	12	169>79	24
Caffeic acid	180.16	179.0344	179>135	16	179>107	24
Ferulic acid	194.18	193.0520	193>134	12	193>178	12
Resveratrol	228.24	227.0678	227>143	28	227>185	20
Apigenin	270.24	269.0452	269>117	44	269>151	28
Kaempferol	286.24	285.0403	285>239	28	285>117	56
Eriodictyol	288.25	287.0793	287>151	12	271>135	28
Catechin	290.27	289.0739	289>245	12	289>203	10
Epicatechin	290.27	289.0742	289>245	12	289>203	10
Quercetin	302.24	301.0100	301>151	20	301>179	20
p-Coumaric acid *O*-glucoside	326.30	325.0932	325>163	10	325>145	10
Gallic acid O-glucoside	332.26	331.0697	331>169	12	331>125	12
Caffeic acid O-glucoside	342.30	341.0896	341>179	20	341>323	10
Chlorogenic acid	354.31	353.0905	353>191	16	353>85	16
Feruloylquinnic acid	368.34	367.1062	367>193	40	367>161	40
Resveratrol O-glucoside	390.38	389.0675	389>227	20	389>185	20
Kaempferol-3-*O*-glucoside	448.38	447.0974	447>284	28	447>255	40
Eriodictyol-7-*O*-glucoside	450.39	449.1084	449>287	12	449>151	36
Catechin O-glucose	452.41	451.1298	451>289	20	451>245	20
EGCG	458.37	457.0700	457>169	16	457>305	20
Quercetin *O*-glucoside	464.38	463.0858	463>299.9	16	463>271	20
Hyperoside	464.38	463.0917	463>299.9	32	463>271	48
Isorhamnetin-3-*O*-glucoside	478.40	477.1014	477>314	32	477>285	40
Procyanidin dimer B2	578.52	577.1389	577>425	12	577>407	28
Kaempferol-3-*O*-rutinoside	594.52	593.1560	593>285	32	593>255	60
Rutin	610.52	609.1508	609>299.9	40	609>271	60
Procyanidin trimer	866.78	865.2041	865>289	20	865>713	20
Cyanidin *O*-arabinoside	419.36	419.0963	419>287	25		
Cyanidin *O*-caffeoylglucose	611.53	611.1601	611>287	25		
Cyanidin *O*-glucose	449.39	449.1076	449>287	25		
Cyanidin-3-*O*-rutinoside	595.53	595.1656	595>287	40		
Delphinidin 3-*O*-rutinoside	611.53	611.1603	611>303	25		
Delphinidin *O*-coumaroylglucose	611.53	611.1391	611>303	25		
Malvidin *O*-coumaroylglucose	639.58	639.1709	639>331	25		
Malvidin-3-O-glucoside	493.44	493.1337	493>331	25		
Pelargonidin O-glucose	433.39	433.1125	433>271	30		
Peonidin-3-O-rutinoside	609.56	609.1813	609>301	30		

[a] Anthocyanins were analysed in the positive ion mode. [b] Molecular weight detected by qTOF

Abbreviations: MW, molecular weight; CE, collision energy.

Table S3: HPLC-ESI-MS/MS method quality parameters for the studied phenolic compounds in sweet cherries.

Compound[a]	RT (min)	Calibration curve	R^2	Linearity (µM)	LOD (nM)	LOQ (nM)
Benzoic acid	9.77	y=304.74x	0.993	0.016-40.943	7.18	23.94
Phloroglucinol	2.80	y=203.80x	0.992	0.016-39.648	0.40	1.33
Protocatechuic acid	3.90	y=11.11x	0.996	0.013-32.442	18.20	60.68
p-Coumaric acid	7.68	y=3567.30x	0.999	0.012-30.458	0.87	2.89
Gallic Acid	3.02	y=11453.00x	0.996	0.012-29.391	0.10	0.33
Caffeic acid	5.85	y=88.55x	0.999	0.011-27.754	4.73	15.76
Ferulic acid	8.32	y=1019.40x	0.996	0.010-25.749	0.43	1.43
Resveratrol	11.71	y=529,84x	0.998	0.009-21.906	0.08	0.28
Apigenin	14.57	y=1950.50x	0.992	0.007-18.502	0.09	0.32
Kaempferol	13.03	y=1489.30x	0.997	0.007-17.468	0.16	0.49
Eriodictyol	12.84	y=1556.40x	0.995	0.007-17.346	0.05	0.15
Catechin	5.13	y=369,62x	0.991	0.007-17.225	0.69	2.29
Epicatechin	6.17	y=500,25x	0.995	0.007-17.225	0.66	2.20
Quercetin	13.25	y=1628.00x	0.996	0.007-16.543	0.05	0.17
Chlorogenic acid	4.80	y=1032.10x	0.999	0.006-14.112	0.32	1.07
Kaempferol-3-*O*-glucoside	9.55	y=1073.20x	0.993	0.004-11.151	0.06	0.19
Eriodictyol-7-*O*-glucoside	8.44	y=1968.60x	0.995	0.004-11.101	0.04	0.15
EGCG	6.40	y=1699,3x	0.999	0.004-10.908	0.05	0.16
Hyperoside	8.37	y=1527.80x	0.994	0.004-10.767	0.04	0.14
Isorhamnetin-3-*O*-glucoside	9.71	y=1058.90x	0.990	0.004-10.451	0.04	0.15
Procyanidin dimer B2	5.63	y=192,39x	0.999	0.003-8.634	0.29	0.96
Kaempferol-3-*O*-rutinoside	9.04	y=976.41x	0.998	0.003-8.410	0.03	0.09
Rutin	8.07	y=825.00x	0.998	0.003-8.190	0.06	0.20
Cyanidin-3-*O*-rutinoside	8.21	y=19.55x	0.998	0.003-3.358	0.88	2.92
Malvidin-3-*O*-glucoside	10.99	y=258.29x	0.999	0.004-4.053	1.13	3.77
Peonidin-3-*O*-rutinoside	10.59	y=118.74x	0.999	0.003-3.281	0.78	2.61

[a] Anthocyanins were analysed in the positive ion mode.

Abbreviations: RT, retention time; R^2, determination coefficient; LOD, limit of detection; LOQ, limit of quantification.
